# Bipolar disorder and substance use: Risk factors and prognosis

**DOI:** 10.1192/j.eurpsy.2023.1472

**Published:** 2023-07-19

**Authors:** M. Fernández Lozano, B. Rodríguez Rodríguez, M. J. Mateos Sexmero, N. Navarro Barriga, C. Vallecillo Adame, C. de Andrés Lobo, T. Jimenez Aparicio, M. Queipo de Llano de la Viuda, G. Guerra Valera, A. A. Gonzaga Ramírez, M. P. Pando Fernández, M. Calvo Valcárcel, M. A. Andreo Vidal, P. Martínez Gimeno

**Affiliations:** Hospital Clínico Universitario de Valladolid, Valladolid, Spain

## Abstract

**Introduction:**

Bipolar disorder comorbidity rates are the highest among the major mental disorders. In addition to other intoxicants, alcohol is the most abused substance because it is socially accepted and can be legally bought and consumed. Estimates are between 40-70% with male predominance, which further influences the severity with a more complicated course of both disorders.

**Objectives:**

The objective of this article is to highlight the impact of substance use on the course and prognosis of bipolar disorder, as well as to make a differential diagnosis of a manic episode in this context.

**Methods:**

Bibliographic review of scientific literature based on a relevant clinical case.

**Results:**

We present the case of a 45-year-old male patient. Single with no children. Unemployed. History of drug use since he was young: alcohol, cannabis and amphetamines. Diagnosed with bipolar disorder in 2012 after a manic episode that required hospital admission. During his evolution he presented two depressive episodes that required psychopharmacological treatment and follow-up by his psychiatrist of reference. Since then, he has been consuming alcohol and amphetamines occasionally, with a gradual increase until it became daily in the last month. He went to the emergency department for psychomotor agitation after being found in the street. He reported feeling threatened by a racial group presenting accelerated speech, insomnia and increased activity.

**Conclusions:**

The presence of substance abuse complicates the clinical presentation, treatment and development of bipolar disorder. It is associated with a worse prognosis with multiple negative consequences including worsening symptom severity, increased risk of suicide and hospitalization, increased medical morbidity and complication of social problems. In addition, this comorbidity delays both the diagnosis and treatment, by masking the symptoms, and making more difficult an adequate differential diagnosis.

**Disclosure of Interest:**

None Declared

